# Preventing hospital falls: feasibility of care workforce redesign to optimise patient falls education

**DOI:** 10.1093/ageing/afad250

**Published:** 2024-01-25

**Authors:** Meg E Morris, Claire Thwaites, Rosalie Lui, Steven M McPhail, Terry Haines, Debra Kiegaldie, Hazel Heng, Louise Shaw, Susan Hammond, Jonathan P McKercher, Matthew Knight, Leeanne M Carey, Richard Gray, Ron Shorr, Anne-Marie Hill

**Affiliations:** Academic and Research Collaborative in Health (ARCH), and Care Economy Research Institute (CERI), La Trobe University, Melbourne, VIC, Australia; Victorian Rehabilitation Centre, Healthscope, Glen Waverley, Melbourne, VIC, Australia; Academic and Research Collaborative in Health (ARCH), and Care Economy Research Institute (CERI), La Trobe University, Melbourne, VIC, Australia; Victorian Rehabilitation Centre, Healthscope, Glen Waverley, Melbourne, VIC, Australia; Victorian Rehabilitation Centre, Healthscope, Glen Waverley, Melbourne, VIC, Australia; Australian Centre for Health Services Innovation and Centre for Healthcare Transformation, School of Public Health and Social Work, Faculty of Health, Queensland University of Technology, Brisbane, QLD, Australia; Digital Health and Informatics Directorate, Metro South Health, Brisbane, QLD, Australia; School of Primary and Allied Health Care, Monash University, Melbourne, VIC, Australia; Faculty of Health Sciences & Community Studies, Holmesglen Institute, Melbourne, VIC, Australia; Eastern Health Clinical School, Monash University, Melbourne, VIC, Australia; Northern Health Academic and Research Collaborative in Health (ARCH), La Trobe University, Melbourne, VIC, Australia; Northern Health, Epping, VIC, Australia; Academic and Research Collaborative in Health (ARCH), and Care Economy Research Institute (CERI), La Trobe University, Melbourne, VIC, Australia; Centre for Digital Transformation of Health, University of Melbourne, Melbourne, VIC, Australia; Victorian Rehabilitation Centre, Healthscope, Glen Waverley, Melbourne, VIC, Australia; Academic and Research Collaborative in Health (ARCH), and Care Economy Research Institute (CERI), La Trobe University, Melbourne, VIC, Australia; Victorian Rehabilitation Centre, Healthscope, Glen Waverley, Melbourne, VIC, Australia; Occupational Therapy, Department of Community and Clinical Health, School of Allied Health, Human Services and Sport, La Trobe University, Melbourne, VIC, Australia; Neurorehabilitation and Recovery, Florey Institute of Neuroscience and Mental Health, Melbourne, VIC, Australia; School of Nursing and Midwifery, La Trobe University, Melbourne, VIC, Australia; Department of Epidemiology, University of Florida, Gainesville, FL, USA; School of Allied Health, Western Australian Centre for Health & Ageing, The University of Western Australia, Perth, WA, Australia

**Keywords:** falls, injurious falls, hospital, care workforce, care economy, safety, quality, public involvement, older people

## Abstract

**Objective:**

To examine the feasibility of using allied health assistants to deliver patient falls prevention education within 48 h after hospital admission.

**Design and setting:**

Feasibility study with hospital patients randomly allocated to usual care or usual care plus additional patient falls prevention education delivered by supervised allied health assistants using an evidence-based scripted conversation and educational pamphlet.

**Participants:**

(i) allied health assistants and (ii) patients admitted to participating hospital wards over a 20-week period.

**Outcomes:**

(i) feasibility of allied health assistant delivery of patient education; (ii) hospital falls per 1,000 bed days; (iii) injurious falls; (iv) number of falls requiring transfer to an acute medical facility.

**Results:**

541 patients participated (median age 81 years); 270 control group and 271 experimental group. Allied health assistants (*n* = 12) delivered scripted education sessions to 254 patients in the experimental group, 97% within 24 h after admission. There were 32 falls in the control group and 22 in the experimental group. The falls rate was 8.07 falls per 1,000 bed days in the control group and 5.69 falls per 1,000 bed days for the experimental group (incidence rate ratio = 0.66 (95% CI 0.32, 1.36; *P* = 0.26)). There were 2.02 injurious falls per 1,000 bed days for the control group and 1.03 for the experimental group. Nine falls (7 control, 2 experimental) required transfer to an acute facility. No adverse events were attributable to the experimental group intervention.

**Conclusions:**

It is feasible and of benefit to supplement usual care with patient education delivered by allied health assistants.

## Key Points

- Hospital falls are frequent and debilitating and many are preventable.
- We assessed the effectiveness of an allied health assistant-led intervention to reduce falls and fall-related injuries using patient education that commenced on day 1 after admission.
- Allied health assistants, under supervision, were able to deliver falls prevention education within 48 h.
- 8.5% of the control group were fallers, compared to 5.5% in the group that received the allied health assistant education.
- Workforce redesign is warranted to reduce the persistent problem of hospital falls.

## Introduction

Falls continue to be a frequent and avoidable problem in hospitals worldwide [1–6]. Falls rates in hospitals range from 3 to 16 per 1,000 bed days [6] and are higher for very old people and those with cognitive impairment, or chronic conditions such as Parkinson’s disease [6–8]. Between 30 and 50% of hospital falls result in physical injuries and over 2,000 hip fractures occur in UK hospitals yearly [9–11]. Hip fractures in hospital result in significantly poorer patient outcomes compared to those occurring in the community [10]. Over 80% of femoral fractures occur the first time a patient falls [10]. In addition, 80% of in-hospital injury deaths are related to unintentional injuries, most commonly from falls [12, 13]. Hospital falls also lead to pain, disability and loss of confidence [2, 3, 5, 14]. Falls in hospital can increase length of hospital stay in older patients [5, 9, 15]. They are predictive of unplanned discharge to aged care homes [10, 16, 17].

Patient education is a key intervention to reduce hospital falls, with level 1A evidence from clinical trials delivered by registered health professionals [4, 15, 18]. Yet, sometimes allied health professionals and nurses are not able to deliver comprehensive patient falls education for many days due to competing demands of hospital roles, staff workforce shortages or lack of ownership over who should provide patient education [19–21]. World falls guidelines stress the importance of providing patients with early falls preventive education [2, 22] with a multi-factorial approach that includes staff education, exercise, safe footwear, assistive devices, environmental modifications, medication management, and optimal management of delirium and dementia [4, 23–25]. Many falls occur within the first 48 h after admission [26, 27].

Due to rapid population ageing across the globe, care workforce redesign is arguably needed to keep older patients safe in hospital [28]. Despite randomised trials of patient education incorporating an individualised falls prevention education intervention being shown to be very effective in hospitals [15, 18], staff do not always implement comprehensive fall education soon after admission [19]. An alternative approach could be to address this gap in care by using assistants, such as allied health assistants, nurse assistants or personal care assistants, to deliver fall preventive education, under the supervision of qualified health professionals [29–31]. This has not previously been reported.

The aim of this study was to investigate the feasibility of care workforce redesign to reduce hospital falls by using trained assistants to deliver evidence-based, scripted patient falls education in addition to usual care within the first 48 h after the patient’s hospital admission. It was hypothesised that providing timely, evidence-based patient education delivered by trained assistants would be feasible and safe.

## Methods

A feasibility trial incorporating a parallel two-group randomised controlled trial was conducted in an Australian hospital that focused on rehabilitation and aged care. The study was prospectively registered with the Australian New Zealand Clinical Trials Registry (ACTRN12622000928718) before the first hospital ward was recruited. It was approved by the La Trobe University Human Ethics Committee (HREC21429). Consumers were engaged at all stages [32].

Patients were allocated to a control group that received usual hospital care or an experimental group that received usual care plus additional patient falls prevention education delivered by trained allied health assistants within 48 h after admission. A waiver of consent was granted allowing data collected from hospital wards to be de-identified. The study met each of the criteria necessary for a waiver of consent as outlined in the Australian NHMRC National Statement [33].

### Participants

Twelve allied health assistants were recruited from existing and newly employed hospital staff. To be included, they needed to provide informed consent, be willing to receive additional falls prevention training and have completed all standard hospital training competencies. The assistants completed online ‘Good Clinical Practice’ research education. Each allied health assistant reported to a supervising physiotherapist, occupational therapist or treating nurse to ensure safety and quality of service delivery.

Falls data were collected from all patients admitted to two participating 30-bed wards at a rehabilitation hospital for 20 consecutive weeks after 1 August 2022. ‘Riskman’ electronic medical records provided data on falls and their consequences, as well as falls profiles such as where and when falls occurred. The participating wards delivered a broad range of services including neurology, orthopaedics, general medical and post-surgical care with 24-h medical coverage and daily rehabilitation.

Patients allocated to the experimental group were excluded from the education intervention if they had significant cognitive impairment (Mini-Mental State Examination (MMSE) score < 24) [34, 35], low English literacy, debilitating pain, a hearing or visual impairment, or if they were in end-of-life care.

### Randomisation and blinding

All allied health assistants delivered the falls education intervention. It was not possible to blind them because they delivered the falls scripts. Staff on the wards were informed that an allied health falls education trial was occurring but were not given further details.

Patients in each ward were individually randomised to either the control or experimental group and were blind to group allocation. The randomisation was completed sequentially in order of admission, 7 days per week. The sequence was concealed using computer-generated, random group allocation into opaque, sequentially numbered, sealed envelopes by a researcher who was not involved with the interventions or assessments. They were opened by the allied health manager who advised assistants and supervisors of group allocation.

### Interventions

The control group received usual care, documented on the Template for Intervention Description and Replication (TIDieR) checklist [36] (Appendix 1). This included individualised, patient risk assessments and comprehensive care delivered by nurses, physiotherapists, occupational therapists and medical practitioners. The timing, content and dosage of comprehensive care was tailored to the needs of each patient and included interventions such as environment modifications, assistive devices, therapeutic exercise, medical management, patient education, footwear, eyewear, diet, management of delirium and dementia, or multi-factorial interventions. Falls prevention pamphlets were available for all staff to use for patient education.

For the experimental group, patients continued to receive usual care plus additional patient falls education delivered by one of 12 trained allied health assistants within the first 48 h following their admission (Appendix 1). Each assistant received a 3-h training programme informed by educational theory and evidence-based educational design [37–39] prior to patient education. This was conducted by educators specialising in health and hospital staff training and included topics such as how to deliver patient-centred falls education, safety procedures and motivational interviewing. Assistants were provided with a purpose-designed falls prevention education script to deliver within the first 48 h after patient admission (Appendix 2). They practised delivering the script to simulated patients. The assistants received additional training from a research officer on the safe and compliant delivery of study protocols and compliance with hospital policies.

The patient falls prevention education involved the assistants initiating a face-to-face discussion with each patient in the first 48 h, guided by the education script. Using motivational interviewing [40], the assistants helped each patient to develop a goal-orientated action plan to reduce the risk of falling. This plan was conveyed to the supervising clinician who documented in the medical record as per usual goal-setting procedures. Each patient in the experimental group was also given a hospital falls prevention pamphlet that aligned with the recommendations in the World Falls Guidelines [2]. The allied health assistants guided patients in opening, reading and storing the pamphlet, for future reference. They discussed the content and answered any questions raised from the pamphlet. Queries raised by the patients or identified by the assistants were referred to the supervisors for further action. After the education on day 1, the allied health assistants had brief conversations throughout the admission and performed a scheduled follow-up with each patient at day 5.

Documentation in the patient file was completed by the assistants after delivering each initial and follow-up session, noting any concerns. In the event of identifying falls risks or perceived health or safety issues, the assistants immediately contacted the supervising health professional responsible for mitigating risks and liaising with patients.

When documented that a patient had moderate to severe cognitive impairment, delirium or dementia, allied health assistants were instructed by their supervisor not to attempt the education and the supervisor treated the patient. For patients with mild cognitive impairment, delivery of the allied health assistant education was attempted.

### Primary outcome

The feasibility of allied health assistant delivery of patient falls education was the primary outcome. Feasibility was quantified by measuring the time from admission to allied health falls education delivery, the number of adverse events in each group, and the estimated cost of allied health assistant service delivery plus the costs of developing and delivering educational resources and training.

### Secondary outcomes

Secondary outcomes were (i) falls rate per 1,000 bed days for the included hospital wards, (ii) injurious falls and (iii) falls requiring transfer to an acute medical facility. Transfer decisions were made by the hospital’s pre-determined policies and the treating medical doctor and team who were not involved in the research trial. De-identified falls and injury data were obtained for all patients consecutively admitted to the participating wards over the 20-week study period. Each fall was identified from the hospital adverse events electronic record and verified by the hospital quality manager. All falls were reported to the local hospital management, a trial Data Safety Monitoring Board and Human Research and Ethics Committee.

### Data analysis

De-identified data were analysed using Stata [41]. The time from patient admission to allied health falls education delivery was categorised as delivered by 24 h from admission, between 25 and 48 h, after 48 h or not at all. The estimated cost of allied health assistant service delivery was determined from hospital pay schedules and associated hours. The number of falls requiring transfer to an acute hospital and the number of serious adverse events were also tabulated (Table 3). Fall rates (all falls and injurious falls) were calculated as falls per 1,000 occupied bed days. Negative binomial regressions rather than regressions from the Poisson family were used to examine differences between groups in both fall rates and injurious fall rates as the standard deviation of inpatient hospital fall count data was greater than the mean (over dispersed). Length of stay (days) was included as the exposure, and these regressions were otherwise unadjusted as we did not expect or observe between group selection or informational bias. The economic evaluation pertained to costs for AHA wages, education and development, educational resources and delivery of allied health assistant (AHA) training.

**Table 1:** Participant characteristics

	Control (*n* = 270)	Experimental (*n* = 271)	Total (*n* = 541)
Age on admission^a^	80.0 (27–98)	81.0 (38–100)	81.0 (27–100)
Male	105	100	205 (38%)
Female	165	171	336 (62%)
**Key reason for admission**			
Fall-related injury	57	61	118 (22%)
Illness or infection	60	47	107 (20%)
Arthritis	59	53	112 (21%)
Cardiopulmonary	34	35	69 (13%)
Chronic pain	18	13	31 (6%)
Gastrointestinal	12	18	30 (6%)
Neurological	8	15	23 (4%)
Cancer	7	9	16 (3%)
Recurrent falls	6	8	14 (3%)
Vascular	8	4	12 (2%)
Cognitive impairment	62	89	151 (28%)
Other	1	8	9 (2%)
Admission length (days)	13 (0–38)	13 (1–34)	13 (0–38)

^a^Data expressed as median (range).

## Results

### Allied health assistant demographics

Twelve allied assistants participated, and all received falls education training. Five had less than 1 year of experience working as an assistant. The median age of assistants was 31 years (21–55) and 92% were female (Table 2).

**Table 2:** Allied health assistant (AHA) data (*n* = 12)

Female assistant	11
Male assistant	1
Age (median, range)	31 (21–55)
Years of experience (median, range)	1 (0–12)
Experimental group patient falls education delivered	254 (94%)
Number of interventions delivered per health assistant (median)	11
Falls education delivered within 24 h of admission	246
Falls education delivered 24–48 h of admission	8
Follow-up	205
**Reasons for AHA education not delivered (*n* = 17)**	
Non-English speaking	8
AHA directed by supervisor not to see patient due to significant cognitive impairment, delirium or dementia	6
Patient discharged on day of admission	1
Patient in contact precautions	2

### Patient demographics

**Figure 1:**
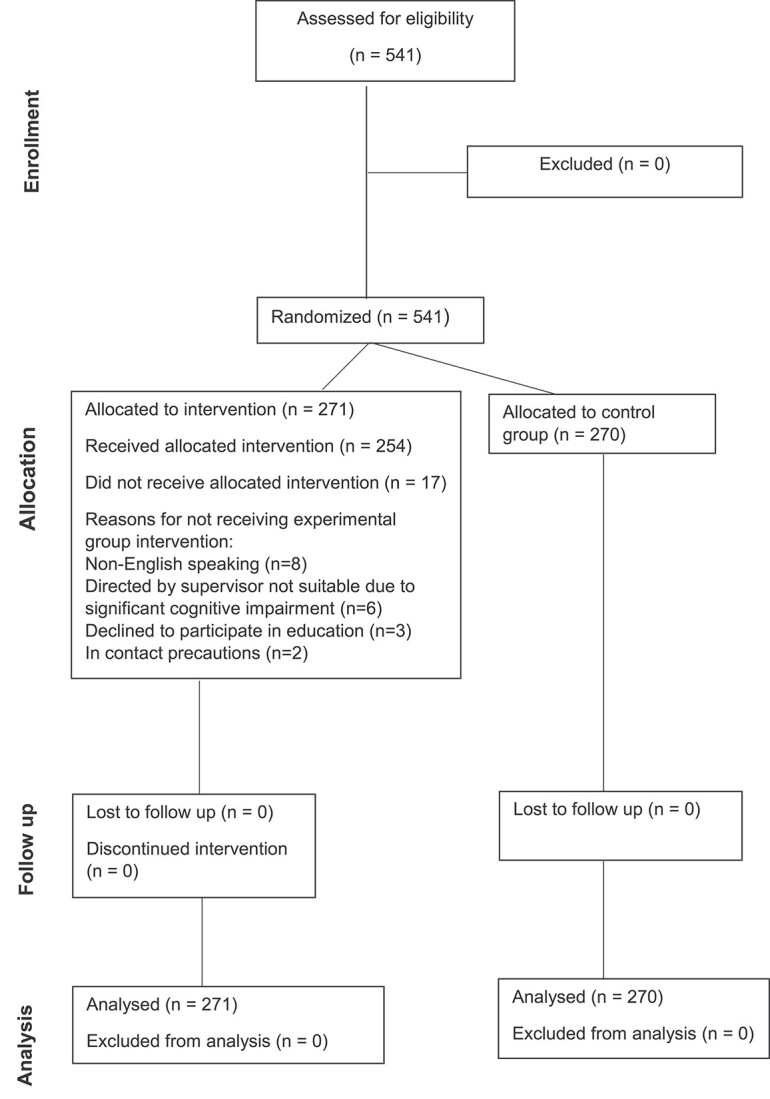
Patient flow through the study.

We included 541 consecutive patients from two private hospital rehabilitation wards. As seen in the CONSORT flow chart [42] (Figure 1), there were 270 patients in the control group and 271 in the experimental group. Each hospital ward had 30 individual patient rooms, with ensuite bathrooms. The average daily occupancy over the 20 weeks of the trial was 29 patients per ward (range 24–30). The median age of the patients in the trial was 81 years and 62% were female (Table 1). Cognitive impairment or diagnosed delirium was present in 28% of participants. The average length of admission was the same for both groups, at 13 days.

### Feasibility of allied health assistant falls education

Education was delivered within 24 h of admission in 97% of experimental group participants and within 48 h for the remaining 3%. Table 2 shows that scripted falls education was delivered by the allied health assistants to 254/271 (94%) of the patients in the experimental group. Eighty-one percent of the experimental group patients had at least one follow-up session with the assistants. Falls and other adverse events are reported in Table 3 and were monitored by weekly data reviews by the hospital and research team and reported to the data safety monitoring committee (DSMC) and ethics committee. There were no adverse events directly attributable to the delivery of the allied health assistant education. No known cases of contamination occurred. The main barriers to allied health education are summarised in Table 2.

**Table 3:** Fall outcomes and adverse events in each group

	Control group	Experimental group	Total
Falls rate/1,000 bed days^a^	8.07	5.69	6.89
Number of falls	32	22	54
Number of participants who fell	23	15	38
Falls with injury	8	4	12
Falls with serious adverse event^b^	1	0	1
Required transfer to acute medical facility	7	2	9

^a^Control group bed days = 7,271; experimental group bed days = 7,284.

^b^Serious adverse event includes falls resulting in major injury with long-term consequences including fractures, brain injury or death.

The average hourly rate for allied health assistants was $41.60 AUD per hour. AHA wages for 3 h of face-to-face patient education plus 1 h for documentation and discussion with supervisors were $166.40 AUD per assistant per patient, for 271 patients ($45,094.40 AUD). Each AHA also attended 8 h of education and training ($3,993.60 AUD). Fees for educators to design the programme, create the lesson plan and teaching resources, and deliver the education with simulated participants were $5,120 AUD. Hence, for the 271 patients in the experimental group, the cost beyond usual hospital service delivery was $54,208 AUD ($200 per patient).

### Falls rates

Over 20 weeks, 32 falls occurred in the control group and 22 falls in the experimental group. The falls rate for the entire sample was 6.89 per 1,000 bed days. The falls rate was 8.07 falls per 1,000 bed days in the control group compared to 5.69 falls per 1,000 bed days for the experimental group. The difference between groups in fall rates expressed as an incident rate ratio was not statistically significant (incidence rate ratio (IRR) = 0.66 (95% CI 0.32, 1.36; *P* = 0.26)). Post hoc analysis indicated that a sample size of 4,294 would be required for 90% power to detect a statistically significant (*P* < 0.05) difference in fall rates consistent with negative binomial regression in this trial, overdispersion coefficient of up to 7.3 and mean patient length of stay of 14.7 days [43].

The total number of patients that fell was 38 (7%). There were 23 fallers in the control group (8.5%) compared to 15 fallers in the experimental group (5.5%). The difference in number of fallers expressed as an odds ratio from negative binomial regression was not statistically significant (OR = 0.63 (0.32; 1.23); *P* = 0.18). There were 2.02 injurious falls per 1,000 bed days for the control group compared to 1.03 injurious falls per 1,000 bed days in the experimental group; the difference between groups expressed as an incident rate ratio was IRR = 0.49 (95% CI 0.13, 1.77; *P* = 0.27). Seven fallers in the control group and two fallers in the experimental group required transfer to an acute medical facility due to injuries associated with the fall. One faller in the control group required surgical repair of a soft tissue injury from the fall. None of the falls during the study resulted in fractures or death (Table 3). There were 6 recurrent fallers (more than 1 fall) in the control group (median 2 falls, range 2–4 falls) and 7 in the experimental group (median 2 falls, range 2–3 falls). The risk of becoming a faller was 0.085 (23 out of 270) in the control group and 0.055 (15 out of 271) in the intervention group, making this an absolute risk reduction of 0.030. The number needed to treat to prevent one person from becoming a faller whilst in hospital was 33.

### Fall profiles in the intervention group

Seven patients in the experimental group fell after receiving the scripted falls education and pamphlet delivered by an allied health assistant. Five of these had mild cognitive impairment or early dementia. Another two in the experimental group fell within 12 h of admission, before they had time to receive the allied health assistant falls education. There were six fallers in the experimental group who did not receive the education following advice from the allied health assistant’s supervisor not to proceed. Table 4 shows that falls mainly occurred in bedrooms (48%) or bathrooms (41%). The majority were unwitnessed falls (66%). As shown in Appendix 3, falls occurred across morning, noon and night, with most between 06:00 and 11:00.

**Table 4:** Characteristics of the fall events in each group

	Control (*n* = 270)	Experimental (*n* = 271)	Total (*n* = 541)
**Location of fall**			
Bedroom	16	11	27
Bathroom	11	10	21
Communal ward space	5	1	6

## Discussion

This trial showed the potential for hospital falls rates and fall-related injuries to be mitigated in some patients using care workforce redesign. It was feasible and safe for supervised assistants to educate hospital patients about how to prevent falling. All fall outcomes (number of falls, number of people falling and injuries) showed trends towards reductions in the intervention group compared to the control group. Despite not reaching statistical significance due to the comparatively small number of falls (54), this was in agreement with prior research showing that education of patients about how to prevent hospital falls can improve outcomes [15, 18].

The strength of the current trial is that principles of workforce redesign [44] were used to overcome this system barrier, to ensure that a trained care worker had ample time, education and resources, to deliver an evidence-based script within the first day. The education aimed to raise patient motivation and to give patients simple goals to reduce their risk of falls and was based on systematically reviewing hospital falls education trials [45]. The mechanism of this education intervention is supported by previous qualitative studies showing that patients are often unaware of their risk of falls, overestimate their ability to mobilise unaided and have limited interaction with staff to gain knowledge about falls prevention in hospital and are not confident to seek help from staff [19, 20, 46].

Clinical trials on hospital falls prevention have traditionally focused on low beds [47], bed alarms [23], chair alarms [48], sock-heel sensors [49], grip socks [50, 51], body sensors [52] and video footage [53]. These strategies have comparatively little success, arguably because they do not involve the patients or families, or because they do not encourage health professionals to spend greater time communicating with patients about safety. Staff communication is an effective means of increasing patients understanding about how to reduce falls and injuries [15, 54, 55]. Once a sensor or video device has been activated, health professionals can have a false sense of security that the patient is safe. However, the sensor alarm is often only activated when the patient actually falls. The longitudinal study by Shorr et al. (2019) [6] on over 26,000 patients showed that divesting from bed alarms did not increase falls yet saved over US$5 million over 2 years.

In the current trial, some patients with mild cognitive impairment benefited from the interaction with an assistant. Previous studies have enrolled patients with minor cognitive impairment (MMSE above 23/30) [15] and shown benefit provided that the conversation is timely and paced appropriately. Carefully structured conversations can also address problems with risk taking [55–57] and improve uptake of falls prevention education [15, 58].

There are several limitations of this feasibility study. It was pragmatic and not powered to detect a significant effect for falls rates. The intervention is suitable for hospitals that employ allied health assistants, limiting the external validity of the results. Only 3% patients in our sample did not have sufficient health literacy or English language to participate, and in other geographic settings or hospital types, this could be higher. We did not provide the education in languages other than English, which is a limitation for scaling up this intervention throughout the healthcare system. Finally, the trial did not set out to determine the best methods to reduce hospital falls in patients with moderate to severe cognitive impairment, delirium or dementia. This is a high priority for future research.

## Conclusions

Workforce redesign can enable timely delivery of evidence-based patient education aimed at reducing falls.

## Supplementary Material

aa-23-1339-File002_afad250

aa-23-1339-File003_afad250

aa-23-1339-File004_afad250
