# Supplementary material for: Preventing hospital falls: feasibility of care workforce redesign to optimise patient falls education
Source: Age Ageing. 2024 Jan 25;53(1):afad250. doi: 10.1093/ageing/afad250 (PMC10811524; doi:10.1093/ageing/afad250)
Supplement: aa-23-1339-File002_afad250 [file aa-23-1339-file002_afad250.docx]

**Preventing Hospital Falls: Feasibility of Care Workforce Redesign to Optimise Patient Falls Education**

**Appendix 1**. Patient education intervention according to TIDieR (Template for Intervention Description and Replication) checklist [36].

| **Item** | **Description** |
| --- | --- |
| 1. Brief name | Patient education to prevent hospital falls. |
| 2. Why | Falls continue to be a significant adverse event in hospitals, resulting in serious injuries and other adverse outcomes [4, 10]. Previous evidence from randomised trials has demonstrated that individualised patient education is an effective intervention to reduce falls [15, 18]. Recently published world fall guidelines and systematic reviews have recommended (Grade IA evidence) that patient education be provided for hospital patients [2-4]. |
| 3. What- materials | Control group: Usual care provision of the hospital falls prevention pamphlet that adhered to world fall guidelines.  Experimental group: A scripted conversation (see Appendix 2 for conversation guide), and the hospital falls prevention pamphlet. The conversation was based on raising patients’ awareness of their risk of falls in hospital and built patients’ motivation and capability to engage in desired fall preventive behaviours. It assisted participants to undertake goal setting and develop a practical plan of action. The content of the scripted conversation was based on world fall guidelines and falls evidence [2, 4, 15, 45]. The design also used concepts of adult learning and motivational interview theory [59-61]. |
| 4. What- procedures | - Control group: Usual care including admission screening of falls risk by nursing, medical and allied health staff. AHAs undertook work with any patients (either intervention or control) according to therapist instructions. AHAs were instructed not to have the scripted conversation with patients in the control group or discuss the pamphlet. - Experimental group: Allied Health Assistants (AHAs) received notification each morning (7 days/ week) of an experimental group participant and discussed if the patient was appropriate to receive education with supervising physiotherapist (PT) or occupational therapist (OT) in relation to the cognitive level of patient, medically able to be approached and appropriate for inclusion. The AHA then visited participants at their bedsides, delivered the education, recorded the delivery in medical records and reported back to their supervisor.   Patient goals were recorded by the AHA and reported back to the supervising PT or OT for documentation in the medical record as per usual goal-setting practice to communicate with the multidisciplinary team.   - Patients identified as having immediate falls risk such as inappropriate footwear, no walking aid or a faulty call bell were immediately referred to the supervising PT or OT and treating nurse. |
| 5. Who - provided | Control group: Usual care by multidisciplinary team of medical, nursing and allied health staff as individually indicated.  Experimental group: AHAs provided all education sessions under the supervision of registered health professionals (OT or PT or nurse). The design of the materials and the training to deliver the intervention was undertaken by researchers with expertise in hospital falls prevention education and health professional education. The AHAs received three hours of face-to-face training at baseline from these experts to deliver the intervention. Training was based on two reviews on hospital falls education, findings from effective education trials and health professional education theory [14, 19, 44, 62, 63]. This included simulation activities (both face-to-face and online) to rehearse the conversation and learn motivational interviewing techniques. |
| 6. How | Control group: Received usual care.  Experimental group: Education was delivered to each patient individually and using a face-to-face format. |
| 7. Where | Control group: Usual care in two rehabilitation wards within one hospital.  Experimental group: The education was delivered in two rehabilitation wards. All patients received the education at their bedside. Patients on the wards had a variety of diagnoses and rehabilitation programs, with varied projected length of stay. |
| 8. When and how much | - Control group: Upon admission, all patients underwent usual care admission procedures of immediate medical and nursing assessment, followed by physiotherapy assessment within 24 hours. Occupational therapy and other allied health assessments occurred according to individual needs. - Experimental group: Within 48 hours of admission, all patients had at least one session of education of approximately 20-30 minutes duration. Patients received at least one follow-up session of approximately 10 minutes on or near the fifth day of admission where the patients’ goals were revisited, and the pamphlet was reviewed. The sessions varied in length dependent on the individual participant needs, with the estimated time being approximately 30 minutes for total contact including initial and follow up visits. The education was delivered daily (7 days/ week) over the 20 weeks of the trial period. |
| 9. Tailoring | Experimental group: All patients received the same pamphlet and AHA interaction. However relevant aspects of the education were tailored according to patients’ feedback during formative discussions. The educator personalised the information provided so it was relevant for that participant and developed appropriate goals to minimise falls risk whilst in hospital. Examples of formulated goals included “I will get help to put on my firm shoes and use the frame to move around and exercise,” “I don’t want to fall again so I will make sure I use the call bell, have my shoes on and use the frame before moving around,” “I have aching legs so I will make sure I use the call bell to get help from the nurses.” If required a patient could receive a third or fourth follow-up session to review their goals. |
| 10. Modifications | Experimental group: No modifications were made to the intervention during the trial. The pre-planned procedure was that if during the education session a participant became distressed or was unable to participate in the education the AHA ceased to continue the education and informed the supervisor. |
| 11. How well (planned) | Experimental group: The intervention fidelity was monitored by the supervising PT or OT who discussed the outcome of interventions with the AHA at normal daily handover times and checked AHA medical record documentation as part of their daily allied health intervention documentation routine. |
| 12. How well (actual) | Experimental group: The intervention occurred as planned with over 93% of patients receiving an initial education session and 80% of patients followed up at least once or twice within their admission. There were 254 initial sessions delivered and 205 follow up sessions. |
