# Supplementary figures and images for: Preventing hospital falls: feasibility of care workforce redesign to optimise patient falls education
Source: Age Ageing. 2024 Jan 25;53(1):afad250. doi: 10.1093/ageing/afad250 (PMC10811524; doi:10.1093/ageing/afad250)

**Appendix 2.** Falls prevention script


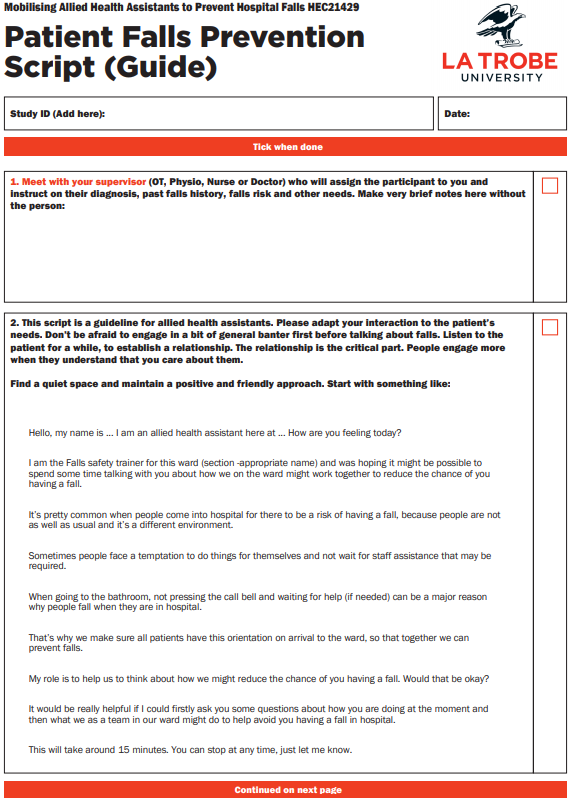


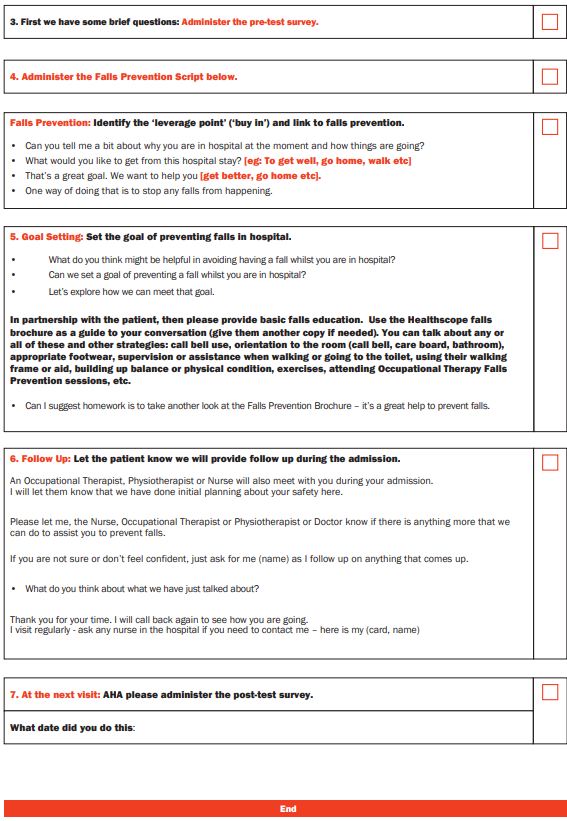

Supplement: aa-23-1339-File003_afad250 [file aa-23-1339-file003_afad250.docx]
