# Supplementary material for: Preventing hospital falls: feasibility of care workforce redesign to optimise patient falls education
Source: Age Ageing. 2024 Jan 25;53(1):afad250. doi: 10.1093/ageing/afad250 (PMC10811524; doi:10.1093/ageing/afad250)
Supplement: aa-23-1339-File004_afad250 [file aa-23-1339-file004_afad250.docx]

**Appendix 3**. Fall recorded time of day (24-hour time)

|  | Number of falls | | |
| --- | --- | --- | --- |
| Time of day | Experimental  (n=22) | Control  (n=32) | Total  (n=54) |
| 00:00-00:59 | 0 | 0 | 0 |
| 01:00-01:59 | 0 | 0 | 0 |
| 02:00-02:59 | 2 | 0 | 2 |
| 03:00-03:59 | 2 | 1 | 3 |
| 04:00-04:59 | 1 | 1 | 2 |
| 05:00-05:59 | 0 | 2 | 2 |
| 06:00-06:59 | 1 | 0 | 1 |
| 07:00-07:59 | 0 | 1 | 1 |
| 08:00-08:59 | 1 | 2 | 3 |
| 09:00-09:59 | 1 | 1 | 2 |
| 10:00-10:59 | 1 | 2 | 3 |
| 11:00-11:59 | 1 | 5 | 6 |
| 12:00-12:59 | 0 | 1 | 1 |
| 13:00-13:59 | 0 | 1 | 1 |
| 14:00-14:59 | 1 | 2 | 3 |
| 15:00-15:59 | 1 | 1 | 2 |
| 16:00-16:59 | 2 | 3 | 5 |
| 17:00-17:59 | 1 | 0 | 1 |
| 18:00-18:59 | 3 | 3 | 6 |
| 19:00-19:59 | 0 | 4 | 4 |
| 20:00-20:59 | 1 | 0 | 1 |
| 21:00-21:59 | 1 | 1 | 2 |
| 22:00-22:59 | 1 | 1 | 2 |
| 23:00-23:59 | 1 | 0 | 1 |
